# Nontuberculous mycobacterial endophthalmitis: case series and review of literature

**DOI:** 10.1186/s12879-020-05606-2

**Published:** 2020-11-23

**Authors:** Warinyupa Pinitpuwadol, Nattaporn Tesavibul, Sutasinee Boonsopon, Darin Sakiyalak, Sucheera Sarunket, Pitipol Choopong

**Affiliations:** Department of Ophthalmology, Faculty of Medicine, Siriraj Hospital, Mahidol University, 2 Wanglang Road, Bangkok Noi, Bangkok, 10700 Thailand

**Keywords:** Atypical mycobacteria, Non-tuberculous mycobacteria, Endophthalmitis, *Mycobacterium haemophilum*, *Mycobacterium fortuitum*, *Mycobacterium abscessus*

## Abstract

**Background:**

To report three cases of nontuberculous mycobacterial (NTM) endophthalmitis following multiple ocular surgeries and to review previous literature in order to study the clinical profile, treatment modalities, and visual outcomes among patients with NTM endophthalmitis.

**Methods:**

Clinical manifestation and management of patients with NTM endophthalmitis in the Department of Ophthalmology, Faculty of Medicine, Siriraj hospital, Mahidol University, Bangkok, Thailand were described. In addition, a review of previously reported cases and case series from MEDLINE, EMBASE, and CENTRAL was performed. The clinical information and type of NTM from the previous studies and our cases were summarized.

**Results:**

We reported three cases of NTM endophthalmitis caused by *M. haemophilum*, *M. fortuitum* and *M. abscessus* and a summarized review of 112 additional cases previously published. Of 115 patients, there were 101 exogenous endophthalmitis (87.8%) and 14 endogenous endophthalmitis (12.2%). The patients’ age ranged from 13 to 89 years with mean of 60.5 ± 17.7 years with no gender predominance. Exogenous endophthalmitis occurred in both healthy and immunocompromised hosts, mainly caused by cataract surgery (67.3%). In contrast, almost all endogenous endophthalmitis patients were immunocompromised. Among all patients, previous history of tuberculosis infection was identified in 4 cases (3.5%). Rapid growing NTMs were responsible for exogenous endophthalmitis, while endogenous endophthalmitis were commonly caused by slow growers. Treatment regimens consisted of macrolides, fluoroquinolones or aminoglycosides, which were continued for up to 12 months. Initial and final vision were generally worse than 6/60.

**Conclusions:**

NTM endophthalmitis is a serious intraocular infection that leads to irreversible loss of vision. The presentation can mimic a chronic recurrent or persistent intraocular inflammation. History of multiple intraocular surgeries or immune-deficiency in patient with chronic panuveitis should raise the practioner’s suspicion of NTM endophthalmitis. Appropriate diagnosis and treatment are important to optimize visual outcome.

**Supplementary Information:**

The online version contains supplementary material available at 10.1186/s12879-020-05606-2.

## Background

Nontuberculous mycobacteria (NTM) are aerobic, gram-positive bacilli found in the soil, dust, and water. NTM infection in human can be both acquired from the environment and nosocomial [1]. Based on in vitro culture properties, NTM have been divided into 4 groups by Runyon in 1959 [2]. The photochromogens (Runyon group I) grow slowly over 2–4 weeks and produce yellow pigment after light exposure. *M. marinum, M. kansasii, M. simiae, M. asiaticum* are majority members in this group. The scotochromogens (Runyon group II) also grow over 2–4 weeks and produce yellow-orange pigment. They include *M. scrofulaceum, M. szulgai, M. gordonae, M. xenophi and M. flavescens*. The nonphotochromogens (Runyon group III) grow over 2–4 weeks without producing pigment. The members in this group are *M. avium, M. malmoense, M. intracellulare, M. terrae, M. haemophilum, M. triviale, M. paratuberculosis, M. gastri, M. nonchromogenicum*. The Rapid growers (Runyon group IV) include 3 subgroups; the *M. fortuitum group*, the *M. chelonae/abscessus group*, and the *M. smegmatis group*. They colony within 5 days and do not produce pigment [2]. NTM ocular infections were first reported in a case of *M. fortuitum* keratitis after corneal foreign body removal in 1965. Since then, the infections have been increasingly reported in a variety of ocular infections range from periocular, conjunctival, scleral, corneal, orbital and intraocular infections [1, 3, 4]. Rapid growers especially *M. chelonae*, *M. fortuitum* and *M. abscessus* are the most common causes of ocular infections and generally associated with poor visual outcomes [4–11].

Endophthalmitis is a serious intraocular infection affecting the vitreous cavity and surrounding tissue. It can be categorized into exogenous or endogenous types. Exogenous endophthalmitis occurs when a pathogen enters the eye by direct inoculation, such as intraocular surgery, penetrating ocular trauma, or periocular tissue infection. On the other hand, endogenous endophthalmitis is a secondary infection in the eye resulting from hematogenous spread from distant infection. The pathogenic microbes are commonly bacteria or fungi; however, mycobacteria can rarely cause endophthalmitis. Being a rare intraocular infection, NTM endophthalmitis is a diagnostic and therapeutic challenge in clinical practice and the prognosis is usually poor accordingly. Herein, we reported three cases of postoperative endophthalmitis caused by different types of NTM at the Department of Ophthalmology, Faculty of Medicine, Siriraj hospital, Mahidol University, Bangkok, Thailand from January 2006 to September 2019. The first case was *M. abscessus* endophthalmitis after a cataract surgery. The second patient developed *M. fortuitum* endophthalmitis after Baerveldt shunt implantation. The third patient was *M. haemophilum* endophthalmitis following multiple trabeculectomy surgeries. The last case was also previously reported in February 2018 [12]. In addition, the literature review and summary of previous NTM endophthalmitis was performed in order to summarize the clinical profile, treatment modalities, and visual outcomes in this rare intraocular infection worldwide.

### Case 1

A 55-year-old man underwent an uneventful cataract surgery with intraocular lens (IOL) implantation of the left eye. One year postoperatively, he experienced painless visual loss in the operated eye. His vision was 6/36 in the left eye. Slit-lamp examination showed ciliary injection, 2+ cells in the anterior chamber, and posterior synechiae. There was white plague deposited on the posterior capsule which was noted as posterior capsule opacity. The fundus examination was normal. There was no other systemic abnormality identified from the physical examination. He had a previous history of sputum smear-negative pulmonary tuberculosis (TB) which was responsive to a 6-month anti-tuberculosis treatment from a local hospital 2 years ago.

Initially, chronic ocular inflammation was suspected so topical corticosteroid was prescribed, and laser capsulotomy was performed. One month later, his vision decreased to hand motion. The anterior chamber showed plasmoid reaction with 4+ cells and a 1.7-mm hypopyon. Fundus examination was obscured by grade 3 vitreous haze. The B-scan ultrasound revealed heterogenous vitreous echogenicity. As a result, pars plana vitrectomy (PPV) with intravitreal injection of vancomycin was performed followed by topical vancomycin, ceftazidime, amikacin and oral ciprofloxacin. The inflammation was gradually improved. Six weeks later, the vitreous culture was identified as *M. abscessus*. The treatment regimen was changed to intravenous injection of cefoxitin and intravitreal amikacin. After 14 days of intravenous antibiotics, the treatment was changed to a 6 month-course of oral clarithromycin and ciprofloxacin. The final best-corrected visual acuity (BCVA) was 6/9 in the left eye and intraocular pressure (IOP) became normal without antiglaucoma medications.

### Case 2

A 61-year-old man with uncontrolled diabetes underwent Baerveldt shunt implantation in his left eye after failures from four trabeculectomy surgeries. One year later, tube shunt exposure was found and successfully repaired with scleral and buccal mucosal graft. One month after the reparation, he developed non-granulomatous anterior uveitis which partially responded to topical prednisolone. The investigations showed positive Quantiferon-TB gold result. He had been treated as TB anterior uveitis with a combination of isoniazid, rifampicin, pyrazinamide and ethambutol for 2 months. The inflammation improved, so the regimen was changed to isoniazid and rifampicin. One months later, he complained of blurred vision and periocular pain in the left eye. The BCVA was 6/30. The anterior segment examination demonstrated marked circumcorneal injection, however there was no sign of blebitis. There was fibrinous material occluded in the tube and 4+ cells in the anterior chamber. Fundus examination revealed prominent anterior vitreous cells and vitreous opacity.

**Fig. 1:**
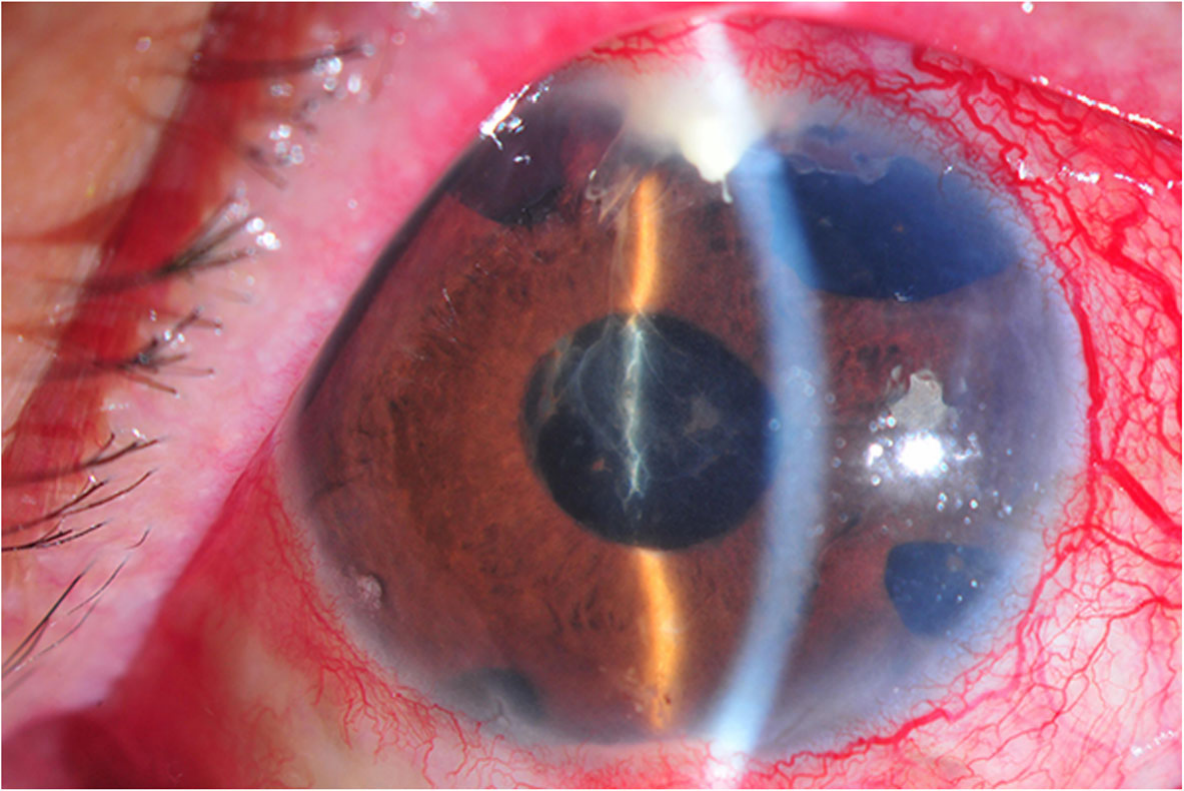
Slit lamp examination of *Mycobacterium fortuitum* endophthalmitis (case 2) demonstrating fibrinous material in the anterior chamber with subconjunctival pus around the tube

Since these findings suggested either endophthalmitis or a progression of ocular tuberculosis, the patient underwent a diagnostic aqueous tapping. Aqueous cultures showed no organisms and the direct polymerase chain reaction (PCR) was negative for TB. Due to persistent intraocular inflammation, PPV was performed followed by multiple intravitreal injections of vancomycin and ceftazidime. Despite these treatments, the inflammation worsened with a development of hypopyon and subconjunctival pus around the tube (Fig. 1). The Baerveldt shunt was removed. Three weeks later, *M. fortuitum* was identified from aqueous cultures in Mycobacteria Growth Indicator Tube (MGIT). The treatment regimen was switched to topical, subconjunctival, and intravitreous amikacin combined with intravenous amikacin, cefoxitin, levofloxacin, and oral clarithromycin. Home medication consisted of oral levofloxacin and clarithromycin for 6 months. The patient showed a good response to the treatment with the final BCVA of 6/18.

### Case 3

A 66-year-old man with uncontrolled diabetes had undergone three trabeculectomies in the left eye; the last surgery performing 5 years ago. Four months before this presentation, he complained of blurred vision and pain in the left eye. The condition was treated with topical and systemic corticosteroids at a local hospital. The intraocular inflammation subsided but recurred after drug discontinuation. After 4-month intermittent treatments, the patient stopped medications and sought for our opinion. At presentation, the left eye showed a poor response to light projection with plasmoid reaction in aqueous, mutton-fat keratic precipitates, and 4+ cells in the anterior chamber. The conjunctiva was injected with three flat trabeculectomy blebs. Severe vitritis with a string-of-pearls appearance was seen from a fundus examination. A diagnosis of severe panuveitis was made. Oral prednisolone was started, and the inflammation subsided.

**Fig. 2:**
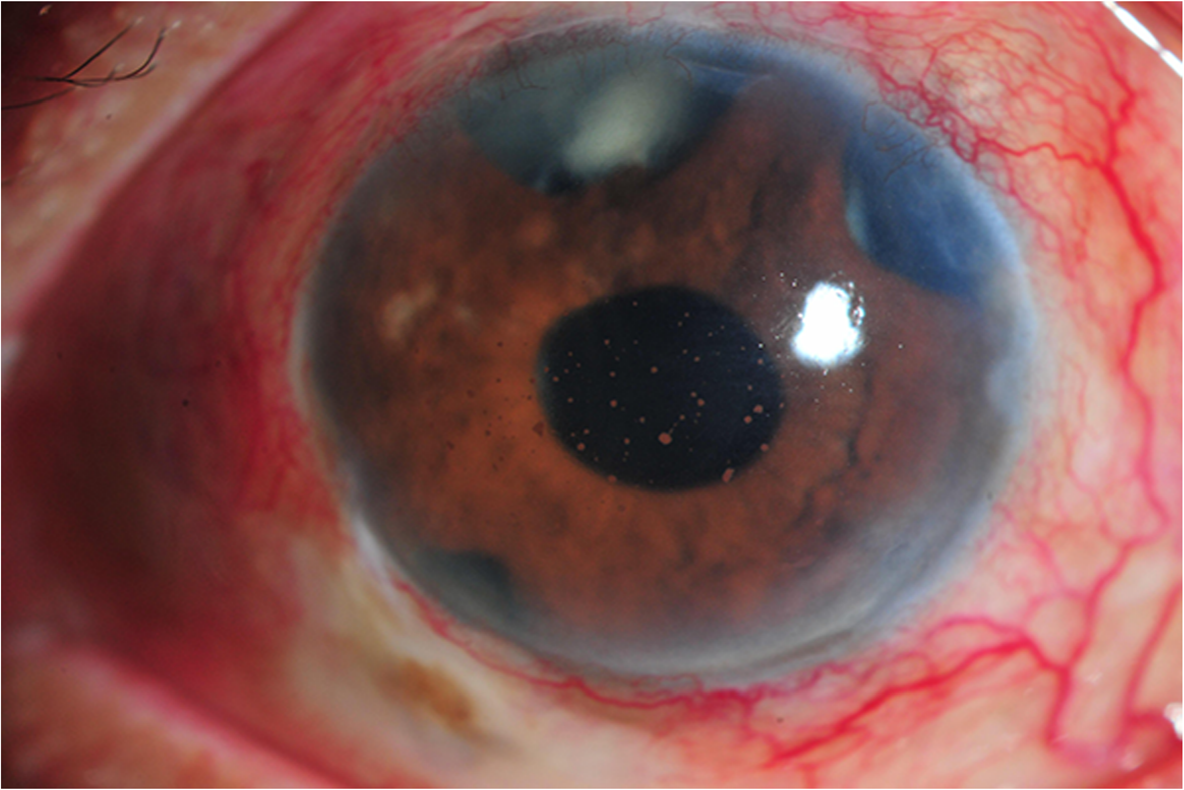
Slit lamp examination of *Mycobacterium haemophilum* endophthalmitis (case 3) at initial presentation demonstrating three flat trabeculectomy blebs with plasmoid aqueous and large mutton fat keratic precipitates in the anterior chamber

**Fig. 3:**
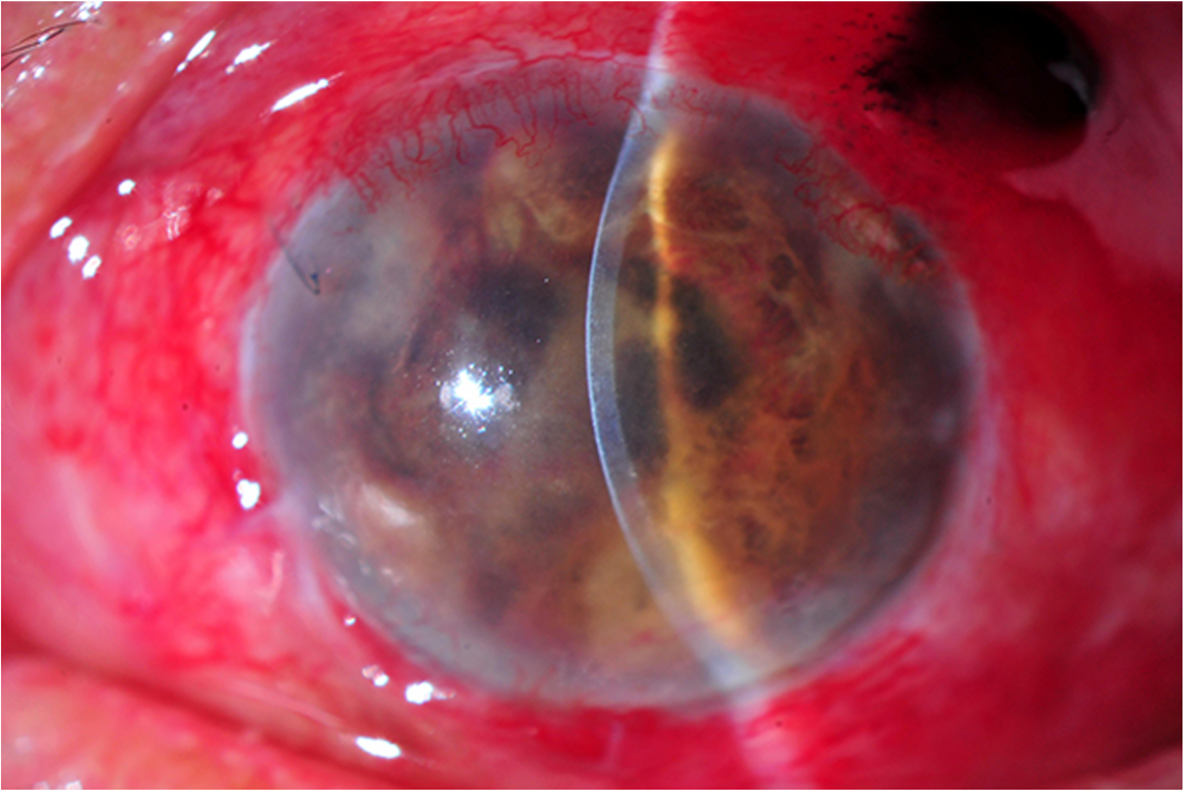
Slit lamp examination of *Mycobacterium haemophilum* endophthalmitis (case 3) at 3 months after initial presentation revealed phthisic eye with prolapsed uveal tissue through a superotemporal area of trabeculectomy wound

One month later, the inferior filtering bleb became inflamed and iris fibrinous membrane and hypopyon were observed in the left eye (Fig. 2). Aqueous and vitreous aspirations were performed along with 2 intravitreal injections of vancomycin and amikacin. Due to unsuccessful results, PPV was carried out. The additional intravenous vancomycin and amikacin were given. Two days later, vitreous staining from vitrectomy sample demonstrated positive for acid-fast bacilli, however the PCR was negative for TB. The treatment was changed to intravenous imipenem, levofloxacin, and amikacin for 2 weeks combined with intravitreal, intracameral, and subconjunctival injections of amikacin and imipenem. The inflammation gradually improved but the vision worsened to no light perception. There was uveal tissue prolapsed through a scleral window of trabeculectomy wound. Eventually, vitreous cultures revealed *M. haemophilum* 3 months later. The treatment was changed to oral azithromycin, doxycycline, and rifampicin for 12 months. Over a follow-up period, the eye gradually became phthisical (Fig. 3).

## Methods

The literature review and summary of previous NTM endophthalmitis was performed in this study. We searched in MEDLINE, EMBASE, and CENTRAL for related articles by using the search terms “atypical mycobacteria”, “non-tuberculous mycobacteria”, “endophthalmitis”, and “intraocular infection”. All reports of patients with a diagnosis of endophthalmitis based on clinical presentation with culture-proven positive for NTM were included. Relevant articles published in English language were extracted and summarized. Patients whose microbiological investigations were negative, and articles with duplicated cases were excluded from data analyses. Our last search was performed in October 2020. We evaluated the methodological quality of published articles using the proposed tool by Murad and colleagues [13] and reported the quality as high, moderate, or low risk of bias. The Preferred Reporting Items for Systematic Reviews and Meta-analyses (PRISMA) guideline was used to report the results of this review.

If available, demographic data including sex, age, systemic diseases, previous ocular trauma and surgery was collected. The information about detectable NTM species, medical and surgical treatment was obtained, as well as the BCVA and complications. All data analyses were performed using SPSS Statistics version 23 (SPSS, Inc.). Demographic data were summarized in descriptive statistics. Categorical data was shown as number and percentage, and continuous data was reported as mean ± standard deviation.

## Results

**Fig. 4:**
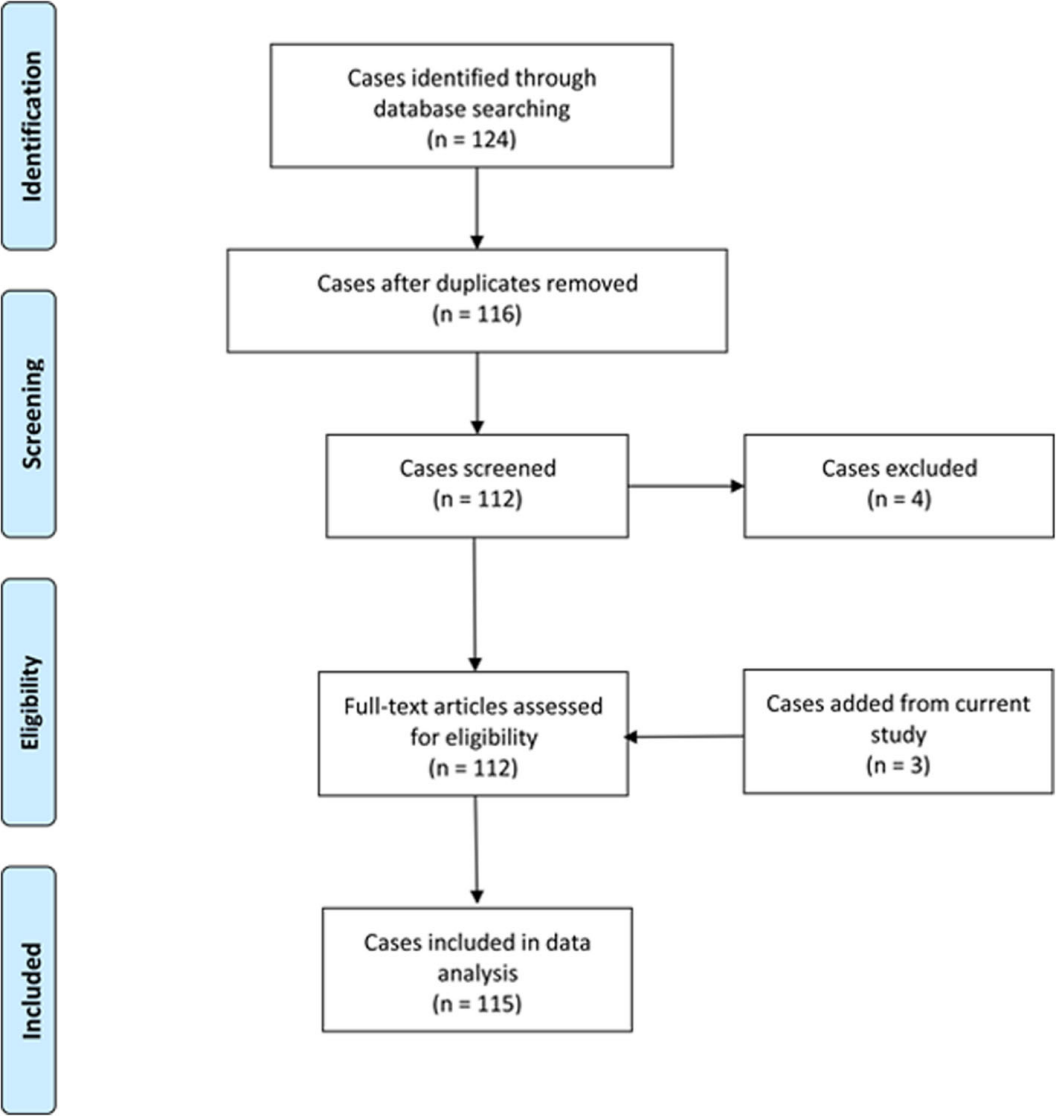
Flow diagram of literature search result

Up to October 2020, there were 112 cases from 51 case reports and case series (Additional files 1 and 2) of culture positive NTM endophthalmitis reported in the literature apart from three cases of this current study [1, 5–7, 14–58]. The search result was illustrated in Fig. 4. Demographic data of all 115 cases were shown in Table 1. The age ranged from 13 to 89 years with mean age of 60.5 ± 17.7 years. There were 54 males (47.0%), 58 females (50.4%), and the data were unavailable in three cases. The common symptoms were pain, decreased vision and redness. The clinical signs are conjunctival injection, hypopyon, anterior chamber inflammation, granulomatous keratic precipitates, vitreous inflammation, and paradoxical deterioration after steroid therapy [3, 4, 36]. Of total, exogenous endophthalmitis and endogenous endophthalmitis occurred in 101 cases (87.8%) and 14 cases (12.2%), respectively.

**Table 1:** Demographic information of patients with Non-tuberculous mycobacterium endophthalmitis

	Exogenous (*n* = 101)	Endogenous (*n* = 14)	Total (*n* = 115)
Sex (%)			
*Male*	46 (45.5)	8 (57.1)	54 (47.0)
*Female*	52 (51.5)	6 (42.9)	58 (50.4)
*not identified*	3 (3.0)	0	3 (2.6)
Mean age in years (SD)	63.2 (16.4)	41.8 (15.9)	60.5 (17.7)
Report of systemic disease (%)			
*No systemic diseases*	33 (32.7)	1 (7.1)	41 (35.7)
*Human Immunodeficiency Virus infection*	0	5 (35.7)	4 (3.5)
*Under immunosuppressants*	9 (8.91)	4 (28.6)	13 (11.3)
*Diabetes mellitus*	14 (13.9)	2 (14.3)	9 (7.8)
*Post-organ transplantation*	1 (1.0)	2 (14.3)	3 (2.6)
*Immunological diseases*	5 (5.0)	1 (7.1)	6 (5.2)
*Cancers*	4 (4.0)	1 (7.1)	5 (4.3)
Previous mycobacterial tuberculosis (%)	2 (2.0)	2 (14.3)	4 (3.5)

*SD* Standard Deviation

In exogenous endophthalmitis cases, the mean age was 63.2 ± 16.4 years (range 13–89 years) and 46 patients (45.5%) were male. Endogenous endophthalmitis patients had the mean age of 41.8 ± 15.9 years (range 17–67 years) and 8 (57.1%) were male.

Exogeneous endophthalmitis occurred in 68 patients underwent cataract surgery (68.3%). Other causes of exogenous endophthalmitis included post-glaucoma drainage implant, post-corneal transplantation, post-intravitreal injection, post-scleral buckling exposure, post-vitrectomy, ocular trauma, corneal ulcer, post-keratoprosthesis, post-trabeculectomy, and post-laser-assisted in situ keratomileusis (LASIK). The presentation started from 1 day to 60 months with a median interval of 1 month after the surgery. From available information on systemic immunological conditions, 23 (22.8%) of exogenous endophthalmitis patients were immunocompromised or had diabetes mellitus. (Tables 1 and 2).

**Table 2:** Primary source of infection of Non-tuberculous mycobacterium endophthalmitis

	Exogenous (*n* = 101)	Endogenous (*n* = 14)
Systemic infections (%)		
*Disseminated NTM infection*	–	4 (30.8)
*Unknown source*	–	7 (50.0)
*Not mentioned*	–	3 (23.1)
Ocular diseases or injuries (%)		
*Cataract surgery*	68 (67.3)	–
*Glaucoma drainage device*	12 (11.9)	–
*Corneal transplantation*	6 (5.9)	–
*Vitrectomy*	3 (3.0)	–
*Intravitreous injection*	2 (2.0)	–
*Scleral buckling*	2 (2.0)	–
*Trauma*	2 (2.0)	–
*Corneal ulcer*	2 (2.0)	–
*Keratoprosthesis*	1 (1.0)	–
*Laser-assisted* in situ *keratomileusis (LASIK)*	1 (1.0)	–
*Trabeculectomy*	1 (1.0)	–
*Not mentioned*	1 (1.0)	–

Of 14 endogenous endophthalmitis patients, 5 (35.7%) were HIV infected and 2 (14.3%) had diabetes mellitus. Four of them received immunosuppressive agents due to autoimmune disease, organ transplantations, or cancers. Only one case was a healthy host. (Table 1) Among endogenous endophthalmitis cases, 4 (30.8%) had a clear evidence of disseminated NTM infection prior to intraocular infection. (Table 2) Others had neither unmentioned systemic infection nor unavailable information. The median duration between the onset of systemic infection and ocular presentation was 3 months which ranged from 15 days to 8 months. From all endophthalmitis cases, there were 4 patients (3.5%) with previous mycobacterial tuberculosis infection. There were one with a lymph node tuberculosis and one with pulmonary tuberculosis (case 1), which had been completely treated 2 years ago. The other two cases had recent ocular (case 2) and disseminated tuberculosis which were undergoing antimycobacterial treatment.

From our reviewed data, positive cultures were found from vitreous (55 cases, 47.8%), aqueous (56 cases, 48.7%), corneal tissue (8 cases, 7.0%), or eviscerated tissue (4 cases, 3.5%). In cases of post-operative endophthalmitis, NTM was also found in the removed intraocular implants (7 cases, 6.1%). The duration between the onsets of ocular infection to final diagnosis varied from 3 days to 12 months with a median of 18 days. The causative organism in exogenous endophthalmitis was mainly identified as rapid growers*; M. chelonae-abscessus group* in 66 patients (65.3%) and *M. fortuitum* in 25 patients (24.8%). On the other hand, the majority of pathogen in endogenous endophthalmitis was slow growers particularly *M. avium* (5 patients, 35.7%). There was 1 patient (7.1%) with mixed infection of *M. fortuitum* and *M. bovis*. The only rapid grower, *M. chelonae,* was identified in 1 patient (7.1%). The details of mycobacterium identification were listed in Table 3.

**Table 3:** Non-tuberculous mycobacterium species identified in endophthalmitis

	Exogenous (*n* = 101)	Endogenous (*n* = 14)
Rapid growers (%)		
*M. chelonae/abscessus* group	66 (65.3)	1 (7.1)
*M. fortuitum*	25 (24.8)	0
*M. manitobense*	2 (2.0)	0
*M. goodie*	1 (1.0)	0
Slow growers (%)		
*M. avium*	0	5 (35.7)
*M. kansasii*	0	1 (7.1)
*M. triplex*	0	1 (7.1)
*M. haemophilum*	1 (1.0)	1 (7.1)
*M. gordonae*	1 (1.0)	0
*M. terrae*	1 (1.0)	0
Mixed infection (%)		
*M. fortuitum* and *M. bovis*	0	1 (7.1)
Unspecified species (%)	4 (4.0)	4 (28.6)

The treatment information was retrieved from 91 exogenous and 9 endogenous endophthalmitis patients. In exogenous endophthalmitis, systemic treatment was given in 66 patients (72.5%) including macrolides (40 patients), aminoglycosides (19 patients), fluoroquinolones (28 patients), cephalosporins (8 patients), beta-lactams (3 patients), vancomycin (3 patients), and clindamycin (1 patient). Local antibiotics (subconjunctival, intracameral, or intravitreal injection) were administered in 81 cases (89.0%) including aminoglycosides (56 patients), vancomycin (65 patients), cephalosporins (47 patients), fluoroquinolones (2 patients), piperacillin-tazobactam (1 patient) and beta-lactams (1 patients). The final regimens were mainly systemic macrolides, fluoroquinolones and aminoglycosides with a duration ranged from 1 week to 12 months.

Of 9 endogenous endophthalmitis patients, systemic treatment was administered in 6 patients (66.7%) included macrolides (4 patients), fluoroquinolones (4 patients), beta-lactams (2 patients), and aminoglycosides (1 patients). Local antibiotics were given in 5 cases (55.6%). All of them received aminoglycosides. Cephalosporins and vancomycin were used in 1 patient each. The final regimen was either systemic macrolides or fluoroquinolones with a duration ranged from 3 weeks to 9 months.

Additional surgeries were performed to get rid of infection in several cases. PPV was done in 68 exogenous endophthalmitis patients (74.7%) and 5 endogenous endophthalmitis patients (55.6%). Enucleation was performed in 18 exogenous endophthalmitis patients (19.8%) and 3 endogenous endophthalmitis patients (23.1%). In exogenous endophthalmitis cases, 39 of them (42.9%) underwent implant removal. (Table 4).

**Table 4:** Treatment summary of Non-tuberculous mycobacterium endophthalmitis

	Exogenous (*n* = 91)	Endogenous (*n* = 9)
Medical treatments (%)		
*Systemic*	66 (72.5)	6 (66.7)
*Local*^a^	81 (89.0)	5 (55.6)
Surgical treatments (%)		
*Pars plana vitrectomy*	68 (74.7)	5 (55.6)
*Enucleation, evisceration*	18 (19.8)	3 (33.3)
*Device removal*	39 (42.9)	–

^a^Local antibiotics; intravitreous, subconjunctival, and intracameral injection

In exogenous endophthalmitis, the initial BCVA were better than 6/60 in 20 patients (24.1%) and worse than 6/60 in 63 patients (75.9%). The final BCVA were better than 6/60 in 21 patients (21.4%) and worse than 6/60 in 77 patients (78.6%). Complications occurred in 30 patients (29.7%) included hypotony or phthisis in 18 patients (17.8%), retinal detachment in 5 patients (5.0%), secondary glaucoma in 3 patients (3.0%), corneal perforation in 3 patients (3.0%), and optic atrophy in 2 patients (2.0%).

In endogenous endophthalmitis, there were 2 patients (28.6%) with BCVA better than 6/60 and 5 patients (71.4%) with BCVA worse than 6/60 at the beginning. At the final visit, 1 patient (10.0%) gained BCVA better than 6/60 and 9 patients (90.0%) had BCVA worse than 6/60. Complications occurred in 5 patients (35.7%); hypotony or phthisis in 3 patients (21.4%), retinal detachment in 1 patient (7.1%), secondary glaucoma in 1 patient (7.1%), and corneal perforation in 1 patient (7.1%). There were no significant differences of initial and final BCVA between both types of NTM endophthalmitis (*p* = 1.000, 0.446, respectively). (Table 5).

**Table 5:** Clinical outcome of Non-tuberculous mycobacterium endophthalmitis

	Exogenous n (%)	Endogenous n (%)
Initial best-corrected visual acuity	*n* = 83	*n* = 7
6/6–6/60	20 (24.1)	2 (28.6)
Worse than 6/60	63 (75.9)	5 (71.4)
Final best-corrected visual acuity	*n* = 98	*n* = 10
6/6–6/60	21 (21.4)	1 (10.0)
Worse than 6/60	77 (78.6)	9 (90.0)
Complications	*n* = 30	*n* = 5
Hypotony, phthisis	18 (17.8)	3 (21.4)
Retinal detachment	5 (5.0)	1 (7.1)
Glaucoma	3 (3.0)	1 (7.1)
Corneal perforation	3 (3.0)	1 (7.1)
Optic atrophy	2 (2.0)	0

## Discussion

Since the first reported case of NTM endophthalmitis in 1973, the organism had been increasingly reported because of improved microbiological diagnostic methods and enlarged immunocompromised hosts in recent years [40].

In this review, endophthalmitis caused by NTM equally occurred in both males and females. The age of onset was approximately in the fourth to sixth decades of life. NTM exogenous endophthalmitis could occur after uneventful ocular surgery, even in healthy hosts. The infection often occurred within 1 month after ocular surgery. Cataract surgery was accounted for the most common procedure related to the infection. On the other hand, NTM endogenous endophthalmitis mainly took place in immunocompromised patients especially those with history of mycobacterial systemic infection.

Overall demographic data did not change from previous reviews of ocular NTM infections. In 2015, Kheir et al reported that NTM endophthalmitis had no gender differences and the median age of presentation was 44 years. Among all exogenous endophthalmitis patients, the infection usually occurred after ocular intervention which 48.6% was cataract surgery with an average time of 11.5 weeks after ocular surgery [3]. In 2012, Moorthy et al reported an average period of 1 month after a procedure, while all endogenous endophthalmitis cases were under immunosuppression [4]. Likewise, Kheir et al reported that more than half (60.0%) of endogenous endophthalmitis cases was associated with immunodeficiency status and previous disseminated mycobacterial infection [3].

The most common causative pathogens of ocular infections are rapidly growing NTMs. *M. abscessus, M. chelonae*, and *M. fortuitum* are responsible for the majority of cases [3, 4]. However, in this review, we found that most of exogenous endophthalmitis cases were caused by rapid growers, while slow growing NTMs were leading causes of endogenous endophthalmitis. According to different natures of NTMs, slow growers are generally found in pulmonary and lymph node diseases [59]. Along with immunocompromised state of hosts leading to susceptibility for systemic spreading, this could explain why slow growing NTMs were apparently related with endogenous endophthalmitis in this study.

In addition to the published literature, there were three exogenous endophthalmitis cases reported from our hospital; *M. abscessus, M. fortuitum, and M. haemophilum* endophthalmitis*.* All of them were male in fifth to sixth decade similar to previously reported cases. Two rapid growers (*M. absecssus* and *M. fortuitum*) were identified early after laser capsulotomy and reparation of exposed tube shunt respectively, while a slow grower NTM, *M. haemophilum,* was found late in a case with multiple trabeculectomies. Despite the types of organism, the onset of these cases was gradual, and the clinical manifestation was subtle at the beginning mimicking chronic uveitis. Their presentation misled the ophthalmologists resulting in delayed investigation and treatment.

*M. abscessus* belongs to *M. Chelonae/abscessus group.* Its identification was relying on identification methods, such as PCR restriction analysis and DNA sequencing [60]. Among the NTMs, *M. chelonae-abscessus group* was known as having the highest resistant rate to antibiotics and anti-tuberculous drugs due to the formation of biofilm [38, 60].

The onset of *M. chelonae/abscessus* endophthalmitis varied from immediate to 3 years after operation and the infection generally presented with chronic granulomatous inflammation. In post-cataract surgery, corneal infiltration around the cataract wound and white plaque-like material on the intraocular lens implant had been observed [5]. The same pattern was observed in our patient. In the previous literature, *M. chelonae/abscessus* group was generally associated with poor visual outcomes [5]. Total of 82.3% of cases ended up with visual impairment, evisceration, enucleation, or phthisis. The poor prognosis is due to delay diagnosis and treatment and the organism’s ability to form a biofilm [1, 6, 9]. The diagnosis of our case was made within 6 weeks after capsulotomy which caused acute active inflammation. This presentation led to early vitrectomy resulting in a successful treatment. From literature review, there were only 11 eyes that showed final BCVA of 6/60 or better [7, 21, 30, 32, 37, 41, 43, 47, 52]. Stewart et al reported a case of *M. abscessus* endophthalmitis with a full visual recovery [50].

*M. fortuitum* endophthalmitis was reported in 26 cases previously. The incubation period ranged from 10 days to 20 months [1, 7, 22, 28, 41, 44, 45, 56]. As a rapid growing nature, our patient had the onset of 1 month postoperatively. The progression of disease and the clinical findings were similar to almost all reported cases. At early stage, with surgical repair of exposed shunt, infectious uveitis could not be excluded. However, the treatment of this patient was misled by false positive Quantiferon-TB test and treated partially with standard anti-tuberculosis drugs. The suspicion of NTM infection was made after clinical worsening despite anti-tuberculosis and anti-bacterial treatment, and the organism was identified 3 weeks after aqueous aspiration. Despite the delay in diagnosis, our patient ended with favorable vision. Differently, from a review, only a quarter of *M. fortuitum* endophthalmitis gained final BCVA of 6/60 or better [7, 28, 41, 44].

*M. haemophilum* endophthalmitis in our patient was previously reported to be the first case of postoperative endophthalmitis in the literature [12]. In 2007, Modi et al reported the first case of disseminated *M. haemophilum* infection in a patient with immunosuppressive medication after a cardiac transplantation [42, 61, 62]. He had the gradual course of ocular infection with multiple skin nodules before turning into a suppurative endophthalmitis and finally enucleated. Our patient also had similar clinical course which ended up with purulent endophthalmitis and visual loss despite combination of intraocular and systemic antimicrobial treatment.

NTM endophthalmitis has a variable of clinical syndrome which can mimic chronic intraocular inflammation and makes initial confusion with other low virulent bacterial and fungal infection [10]. Misdiagnosis with other organisms has been commonly reported before the final diagnosis [34]. Clinical suspicion of NTM infections is necessary especially in immunocompromised patients, with chronic granulomatous intraocular inflammation and intermittent response with anti-inflammatory drugs. Repeat vitreous and aqueous cultures are required to identify the causative organism in which determines the choice of antibiotics. The treatment of NTM endophthalmitis includes the combination of local and systemic antibiotic therapy with or without surgical removal of the implants. The patient who does not response to medical treatment or has an ocular implant is considered surgical therapeutic interventions [3, 10].

Specific guidelines for antibiotics and duration of treatment are still unestablished. The regimen is based on drug sensitivity information and clinical response. Standard anti-tuberculosis drugs such as isoniazid, rifabutin, rifampin, ethambutol, and streptomycin are commonly prescribed. However, NTM are often resistant to these regimens and alternative antimicrobials have emerging roles for NTMs infection due to the potency, pharmacokinetic property, and safety. Especially among rapidly growing NTM, a combination of aminoglycosides, fluoroquinolones and macrolides has shown successful outcome [3, 8, 10]. Griffith et al recommended the treatment for *M. abscessus* skin and soft tissue infections, based on in vitro susceptibility studies, should be a combination of macrolide and one or more medications including amikacin, cefoxitin, or imipenem for 4–6 months of therapy [63]. Nevertheless, Pasipanodya et al showed poor clinical outcomes of macrolide-containing regimen in *M. abscessus* pulmonary infections, defined by sustained sputum culture conversion and recurrent rates [64]. The results from this meta-analysis were consistent with previous in vitro studies which reported that macrolides showed poor kill rates, even at maximal drug concentrations [65]. On the other hand, slow growers are more sensitive to most of anti-tuberculosis drugs [4]. Thus, further studies of treatment regimen are still needed to gain the most favorable outcomes.

There are some mentionable limitations. As NTM endophthalmitis is an uncommon condition, this review was a collection of case reports and case series. Nevertheless, we summarized all available information and demonstrated the characteristics as well as the causative pathogens and the outcomes of NTM endophthalmitis. Lack of required details from the literature review and inaccessibility of non-English data could miss complete information.

## Conclusions

NTM endophthalmitis is rare but can lead to vision-threatening complications. It can imitate chronic intraocular inflammation, so it usually is misdiagnosed as inflammatory uveitis or endophthalmitis caused by other common pathogens. Although the prognosis for NTM endophthalmitis is poor despite invasive treatment, appropriate samples for microbiological identification and suitable treatment regimen are required for preventing the infections from progression to detrimental outcomes. The recommended therapy is a combination of two or more antibiotics based on culture susceptibility. Surgical intervention can be done in case of failure medical treatment or infection control. However, the precise diagnostic yields and therapeutic managements for NTM are still challenging.

## Supplementary Information

**Additional files 1:** **Appendix Table S1**: Characteristics of published reports of culture-proven non-tuberculous mycobacterial endophthalmitis.

**Additional files 2:** **Appendix Table S2**. Data summary of published reports on non-tuberculous mycobacterial endophthalmitis.

## Data Availability

The data that support the findings of this study are available from the corresponding author (PC) upon reasonable request.

## Abbreviations

NTM: Non-tuberculous mycobacteria
*M. haemophilum*: *Mycobacterium haemophilum*
*M. fortuitum*: *Mycobacterium fortuitum*
*M. abscessus*: *Mycobacterium abscessus*
TB: Tuberculosis
IOL: Intraocular lens
IOP: Intraocular pressure
HIV: Human immunodeficiency virus
DM: Diabetes mellitus
LASIK: Laser-assisted in situ keratomileusis
PPV: Pars plana vitrectomy
PCR: Polymerase chain reaction
BCVA: Best-corrected visual acuity
MGIT: Mycobacteria Growth Indicator Tube
