# Supplementary material for: Nontuberculous mycobacterial endophthalmitis: case series and review of literature
Source: BMC Infect Dis. 2020 Nov 23;20:877. doi: 10.1186/s12879-020-05606-2 (PMC7685667; doi:10.1186/s12879-020-05606-2)
Supplement: Supplementary file 1 — Additional files 1: Appendix Table S1: Characteristics of published reports of culture-proven non-tuberculous mycobacterial endophthalmitis. [file 12879_2020_5606_MOESM1_ESM.docx]

Appendix table 1: Characteristics of published reports of culture-proven non-tuberculous mycobacterial endophthalmitis.

| Year | First author | Study period | Country | Study design | Number of NTM cases (n/N) | Risk of bias |
| --- | --- | --- | --- | --- | --- | --- |
| 1973 | Wasserman et al. | 1970 | NY, USA | Case report | 1 | moderate |
| 1989 | Roussel et al. | - | FL, USA | Case series | 2/2 | low |
| 1989 | Ambler et al. | - | OH, USA | Case report | 1 | high |
| 1990 | Cohen et al. | - | MA, USA | Case report | 1 | high |
| 1995 | Abu El-Asrar et al. | - | Saudi Arabia | Case report | 1 | high |
| 1996 | Mutyala et al. | - | MN, USA | Case report | 1 | moderate |
| 1996 | Grenzbach et al. | - | Germany | Case report | 1 | high |
| 1996 | Valenton et al. | - | Philippines | Case report | 1 | high |
| 1998 | Rosenbaum et al. | - | NY, USA | Case report | 1 | high |
| 1999 | Uy et al. | - | MA, USA | Case report | 1 | moderate |
| 2000 | Ramaswamy et al. | 1999 | South India | Case report | 1 | moderate |
| 2001 | Gedde et al. | 1987-1999 | FL, USA | Case series | 1/4 | high |
| 2002 | Gobels et al. | 2002 | Germany | Case report | 1 | high |
| 2002 | Stephenson et al. | - | TN, USA | Case report | 1 | high |
| 2003 | Scott et al. | 1980-2001 | FL, USA | Case series | 5/5 | moderate |
| 2003 | Wilhelmus et al. | 1989 | TX, USA | Case report | 1 | high |
| 2003 | Marin-Casanova et al. | - | Spain | Case report | 1 | high |
| 2003 | Sungkanuparph et al. | 1993-1999 | Thailand | Case series | 2/20 | high |
| 2003 | Benz et al. | - | FL, USA | Case report | 1 | high |
| 2004 | Lalitha et al. | 2000-2003 | South India | Case series | 6/18 | high |
| 2004 | Benz et al. | 1996-2001 | FL, USA | Case series | 3/313 | moderate |
| 2005 | Spencer et al. | - | UT, USA | Case report | 1 | high |
| 2006 | Stewart et al. | - | FL, USA | Case report | 1 | moderate |
| 2006 | Matieli et al. | 2002 | Brazil | Case report | 1 | high |
| 2007 | Modi et al. | - | IL, USA | Case report | 1 | low |
| 2007 | Palani et al. | - | South India | Case series | 3/3 | high |
| 2008 | Jain et al. | - | South India | Case series | 1/2 | high |
| 2009 | Durand et al. | 1990-2006 | MA, USA | Case series | 1/18 | high |
| 2010 | Chang et al. | 2008 | FL, USA | Case report | 1 | high |
| 2011 | Sinawat et al. | - | Thailand | Case report | 1 | moderate |
| 2011 | Shirodkar et al. | 2000-2009 | FL, USA | Case series | 2/118 | moderate |
| 2012 | Henry et al. | 1995-2009 | FL, USA | Case series | 1/49 | moderate |
| 2013 | Couto et al. | - | Argentina | Case report | 1 | moderate |
| 2012 | Deobhakta et al. | 1999-2011 | FL, USA | Case series | 1/9 | high |
| 2013 | Rao et al. | - | East India | Case report | 1 | high |
| 2013 | Rolfe et al. | - | FL, USA | Case report | 1 | moderate |
| 2014 | Mohan et al. | 2007-2012 | East India | Case series | 1/24 | high |
| 2014 | Venkateswaran et al. | - | NY, USA | Case report | 1 | moderate |
| 2015 | Medina Mendez et al. | 1999-2014 | FL, USA | Case series | 2/13 | high |
| 2016 | Paulose et al. | 2004-2015 | South India | Case series | 5/5 | high |
| 2016 | Shah et al. | 1990-2014 | FL, USA | Case series | 19/19 | moderate |
| 2016 | Hung et al. | - | Taiwan | Case series | 2/2 | moderate |
| 2016 | Barkmeier et al. | - | MN, USA | Case series | 2/2 | high |
| 2016 | Dave et al. | - | South India | Case report | 1 | moderate |
| 2017 | Sharma et al. | - | North India | Case report | 1 | moderate |
| 2018 | Hsu et al. | 2014 | Taiwan | Case series | 9/9 | moderate |
| 2018 | Hung et al. | 2014-2016 | Taiwan | Case series | 12/12 | moderate |
| 2019 | Di et al. | 2008-2017 | China | Case series | 15/15 | high |
| 2020 | Suganeswari et al. | - | South India | Case report | 1 | moderate |
| 2020 | Sharma et al. | - | North India | Case series | 1/4 | high |

NTM non-tuberculous mycobacterium
