# Supplementary material for: Nontuberculous mycobacterial endophthalmitis: case series and review of literature
Source: BMC Infect Dis. 2020 Nov 23;20:877. doi: 10.1186/s12879-020-05606-2 (PMC7685667; doi:10.1186/s12879-020-05606-2)
Supplement: Supplementary file 2 — Additional files 2: Appendix Table S2. Data summary of published reports on non-tuberculous mycobacterial endophthalmitis. [file 12879_2020_5606_MOESM2_ESM.docx]

Appendix table 2. Data summary of published reports on non-tuberculous mycobacterial endophthalmitis.

| Reference (n) | Patient characteristics (sex/age/underlying conditions) | Type  (n) | Pathogenic organisms (n) | Cause (n) | Drug sensitivity (n) | Intraocular medications | Systemic medications | PPV | Eyeball removal | Initial BCVA worse than 6/60 | Final BCVA worse than 6/60 |
| --- | --- | --- | --- | --- | --- | --- | --- | --- | --- | --- | --- |
| Wasserman,  1973 (1) | F/63  leukemia | Endogenous | *M. avium* | Sepsis | - | - | R, cycloserine | no | yes | no | yes |
| Roussel,  1989 (2)^a^ | F/85, M/77 | Exogenous (2) | NTM gr IV (1),  *M. chelonae* (1) | Cataract surgery | A | 2/2 IVT (A) | 2/2 (Ce2, TS)  8 weeks | yes | no | 1/2 | 1/2 |
| Ambler,  1989 (1) | M/67  SOT, IMT | Endogenous | *M. chelonae* | Osteomyelitis of calcanei | A, M, Ca | IVT (A, V) | A, M, Ca | yes | no | yes | - |
| Cohen,  1990 (1) | M/27  AIDS | Endogenous | *M. avium* | Disseminated infection | - | - | A, Ce1 | - | no | - | - |
| Abu El-Asrar, 1995 (1) | M/65  DM | Exogenous | *M. chelonae/*  *abscessus* | Cataract surgery | A, M, R, Ce2 | IVT (A, V) | A, V  2 weeks | yes | no | yes | yes |
| Mutyala,  1996 (1) | F/89 | Exogenous | *M. fortuitum* | Cataract surgery | A, F, Ca | IVT (A, V) | A, T  4 weeks | yes | no | no | yes |
| Grenzbach,  1996 (1) | M/60 | unclassified | NTM | - | - | - | A, Ce3, Cl, P | yes | yes | - | yes |
| Valenton,  1996 (1)^b^ | M/76 | Exogenous | *M. fortuitum* | Cataract surgery | - | IVT (A) | - | yes | no | - | 20/70 |
| Rosenbaum,  1998 (1) | F/46  AIDS | Endogenous | *M. avium* | Disseminated infection | - | IVT (A) | A, F, HRZE 12 weeks | no | yes | yes | yes |
| Uy,  1999 (1) | M/19  SJS, IMT | Exogenous | *M. abscessus* | Penetrating keratoplasty | A, M | IVT (A) | M  16 weeks | yes | no | yes | yes |
| Ramaswamy, 2000 (1) | F/66 | Exogenous | *M. chelonae/*  *abscessus* | Cataract surgery | - | IVT (A, V) | A, HRZ  6 weeks | yes | yes | no | yes |
| Gedde,  2001 (1) | F/70 | Exogenous | *M. chelonae* | Penetrating keratoplasty | - | IVT (Ce1, Ce3) | M | no | no | yes | yes |
| Gobels,  2002 (1) | M/32  AIDS, TBLN (treated) | Endogenous | *M. kansasii* | - | H, R, E | no | H, R, E | no | yes | - | yes |
| Stephenson,  2002 (1) | F | Exogenous | *M. terrae* | LASIK | - | - | - | yes | no | yes | yes |
| Scott,  2003 (5) | 1 F, 4 M  age 62-77 | Exogenous (5) | *M. chelonae* | Cataract surgery (3), Corneal ulcer (1), IVT (1) | M (5),  A (2) | 3/5 IVT (A, M, Ca) | 2/5 (R, M)  4-28 weeks | 4/5 | 1/5 | 3/5 | 5/5 |
| Wilhelmus,  2003 (1) | F/80 | Exogenous | *M. chelonae* | Cataract surgery | A, M | IVT (A) | M  8 weeks | yes | no | - | yes |
| Marin-Casanova, 2003 (1) | F/65 | Exogenous | *M. abscessus* | Cataract surgery | A, M | IVT (A, Ce3) | M  4 weeks | yes | no | no | no |
| Sungkanuparph, 2003 (2) | F/24, F/66 | Exogenous (2) | *M. chelonae/*  *abscessus* | Eye injury | - | - | 1/2 (T) | - | 1/2 | - | 1/2 |
| Benz,  2003 (1) | M/62  DM | Exogenous | *M. chelonae* | IVT | A, M | IVT (A, V) | M  12 weeks | yes | yes | no | yes |
| Lalitha,  2004 (6) | 5 F, 1 M  age 17-71 | Endogenous (3)  Exogenous (3) | NTM (5),  *M. chelonae/*  *abscessus* (1) | Disseminated infection (3), Cataract surgery (3) | - | - | - | no | 2/6 | - | 6/6 |
| Benz,  2004 (3) ^c^ | M/33, M/62  DM (1) | Endogenous (1)  Exogenous (1) | *M. chelonae* (1),  *M. triplex* (1) | IVT (1) | - | 1/3 IVT (A, V) | 1/3 M  12 weeks | 1/3 | 1/3 | - | 1/3 |
| Spencer,  2005 (1) | M/67 | Exogenous | *M. goodii* | Cataract surgery | A | IVT (A) | no | yes | no | yes | no |
| Stewart,  2006 (1) | M/58  AI, IMT | Exogenous | *M. chelonae/ abscessus group* | Cataract surgery | A, M, Linezolid | IVT (A) | Ca, M  24 weeks | yes | no | no | no |
| Matieli,  2006 (1) | F/76 | Exogenous | *M. abscessus* | Cataract surgery | A, M, | IVT (A) | M  16 weeks | yes | no | yes | yes |
| Modi,  2007 (1) | M/66  SOT, IMT | Endogenous | *M. haemophilum* | Skin abscess | R, T, F, M | IVT (A) | R, T, F, M  40 weeks | yes | yes | no | yes |
| Palani,  2007 (3) | 1 F, 2 M  age 58-72 | Exogenous (3) | *M. abscessus* (2),  *M. fortuitum* (1) | Cataract surgery | A (3), F (2) | 1/3 IVT (A) | - | - | 1/3 | - | 2 |
| Jain,  2008 (1) | F/55 | Exogenous | *M. chelonae* | Cataract surgery | - | IVT (A, V) | M  1 week | yes | no | no | no |
| Durand,  2008 (1) | M/77  SJS | Exogenous | *M. abscessus* | Type 2 Boston keratoprosthesis | - | IVT | - | yes | no | - | LP |
| Chang,  2010 (1) | F/88 | Exogenous | *M. abscessus* | DSEK | A, Ce2, F | IVT (Ce3, V) | no | yes | no | no | no |
| Sinawat,  2011 (1) | F/47  Sweet’s syndrome | Endogenous | NTM gr IV | Disseminated lymphadenitis | TS, Ca | no | Ca, M, F  9 weeks | no | no | yes | no |
| Shirodkar,  2011 (2)^d^ | F/53, F/69  Immunosuppressed (1) | Exogenous (2) | *M. chelonae/ abscessus group* (2) | Cataract surgery | - | 2/2 IVT (A, Ce3, V) | 2/2 (M , R)  1-4 weeks | 1/2 | 1/2 | 1/2 | 1/2 |
| Henry,  2012 (1) | M/26 | Exogenous | *M. chelonae* | LASIK | A | - | - | - | - | HM | no |
| Couto,  2013 (1) | F/34 | Exogenous | *M. gordonae* | Phakic IOL implant | H, R, M, A, F, TS | IC (F) | E, F, R  16 weeks | no | no | no | no |
| Deobhakta,  2012 (1)^e^ | - | Exogenous | *M. fortuitum* | GDI | - | IVT Ce3, V | - | - | - | - | 20/50 |
| Rao,  2013 (1) | M/13 | Exogenous | *M. fortuitum* | GDI | A, Ce1, F | IVT (A, Ce1) | no | - | no | - | yes |
| Rolfe,  2013 (1) | F/56  DM | Exogenous | *M. abscessus* | Traumatic eye with prosthesis | A, M | no | A, C, M  24 weeks | no | yes | yes | yes |
| Mohan,  2014 (1) | M/51 | Exogenous | *M. chelonae* | Scleral buckle removal | A | IVT (A) | - | - | no | no | no |
| Venkateswaran, 2014 (1) | M/68  leukemia | Exogenous | *M. chelonae* | Corneal ulcer | A | IVT (A, Ce3, V, M) | no | yes | no | - | no |
| Medina Mendez,  2015 (2)^f^ | - | Exogenous (2) | *M. chelonae* (1),  *M. fortuitum* (1) | GDI | - | 2/2 IVT (Ce3, V) | - | - | - | 20/50 1 | 0/2 |
| Paulose,  2016 (5) | 3 F, 2 M  age 32-69 | Exogenous (5) | *M. chelonae* (3),  *M. manitobense* (1),  *M. fortuitum* (1) | Cataract surgery (3), PPV (1), DSEK (1) | A (5),  F (4), V (2) | 5/5 IVT (A, Ce3, V) | - | 4/5 | 1/5 | 5/5 | 4/5 |
| Shah,  2016 (19) | 9 F, 10 M  age 23-86  DM (3), cancer (4), IMT (3), SOT (1), AIDS (1) | Endogenous (2)  Exogenous (17) | *M. chelonae* (7),  *M. chelonae/ abscessus*  (7),  *M. fortuitum* (3),  *M. triplex* (1),  *M. avium* (1) | Cataract surgery (7), GDI (6), IVT (2), PPV (1), Scleral buckle removal (1) Dissiminated infection (2) | A (14), F (3), M (12), Ce2 (2), Linezolid (4) | 14/19 IVT (A, Ce3, V) | 12/19 (M, A, R, F)  1-4 weeks | 8/19 | 4/19 | 13/19 | 14/19 |
| Hung,  2016 (2) | F/13, M/67 | Exogenous (2) | *M. abscessus* (2) | Cataract surgery (2) | - | 2/2 IVT A, Ce3, V) | 2/2 (V, Ce3, M, F) | 2/2 | 0/2 | 2/2 | 2/ 2 |
| Barkmeier,  2016 (2) | F/74, M/72 | Exogenous (2) | *M. chelonae* (2) | Corneal ulcer (1), GDI (1) | - | 2/2 IVT (A) | 2/2 (M, Tigecycline) 24-48 weeks | 2/2 | 0/2 | 1/2 | 0/2 |
| Dave,  2016 (1) | M/50  liver cirrhosis | Exogenous | *M. manitobense* | Cataract surgery | A, Ce1, Ce2, F, V | IVT (A, Ce3, V) | F | yes | no | yes | no |
| Sharma,  2017 (1) | M/38  IMT, TB lung (treating) | Endogenous | *M. fortuitum + M. bovis* | Disseminated infection | - | no | F, H, R, Z, E  48 weeks | yes | no | yes | yes |
| Hsu,  2018 (9) | 7F, 2M  age 59-82  DM (1) | Exogenous (9) | *M. chelonae/ abscessus group* (9) | Cataract surgery (9) | A | 9/9 IVT (A, V) | 9/9 (A, T, M)  12-24 weeks | 9/9 | 2/9 | 5/9 | 9/9 |
| Hung,  2018 (12) | 7F, 5M  age 13-86 | Exogenous (12) | *M. abscessus* (12) | Cataract surgery | A (8), Ca (2), Ce2 (1) | 12/12 IVT (A, Ce3, V) | 11/12 (A, Ce2, Ce3, F, M, R, T, V) 1-12 weeks | 7/12 | 5/12 | 10/12 | 12/12 |
| Di,  2019 (15) | 8F, 7M  age 46-84  DM (7) | Exogenous (15) | *M. fortuitum* (15) | Cataract surgery | - | IVT (Ce3, V) | F 1 week | 15/15 | 1/15 | 15/15 | 15/15 |
| Suganeswari,  2020 (1) | F/28 | Exogenous | *M. abscessus* | PPV | Pi | IVT (Ce3, V, Pi) | - | no | no | yes | yes |
| Sharma,  2020 (1) | M/30  HIV | Endogenous | *M. avium* | - | - | IVT (Ce3, V) | - | yes | no | yes | no |

BCVA best-corrected visual acuity; F Female; M Male; SOT solid organ transplanted; IMT immunosuppressive therapy; AIDS acquired immunodeficiency syndrome; DM diabetes mellitus; SJS Steven-Johnson syndrome; TBLN tuberculous lymphadenopathy; AI autoimmune disease; TB tuberculosis; NTM non-tuberculous mycobacteria; NHL non-Hodgkin lymphoma

LASIK laser-assisted in situ keratomileusis; IVT intravitreous injection; IC intracameral injection; PPV pars plana vitrectomy; DSEK Descemet Stripping Endothelial Keratoplasty; IOL intraocular lens; GDI glaucoma drainage implantation;

A=Aminoglycosides; Ca=Carbapenems; Ce1=1^st^ gen Cephalosporins; Ce2 D=2^nd^ gen Cephalosporins; Ce3=3^rd^ gen Cephalosporins; Cl=Clindamycin; E=Ethambuthol; F=Fluoroquinolones; H=Isoniazid; M=Macrolides; P=Penicillin; Pi=Piperacillin-tazobactam; R=Rifampicin, Rifampin; T=Tetracyclines; TS = Trimetroprim/Sulfamethoxazole; V=Vancomycin; Z=Pyrazinamide

^a^ Data gathered from Scott, 2003, and Moorthy, 2012

^b^ Data gathered from Moorthy, 2012

^c^ Data gathered from Benz, 2003 and Shah, 2016

^d^ Data gathered from Shah, 2016

^e^ Data gathered from Medina Mendez, 2015

^f^ Data gathered from Deobhakta, 2012
